# The SQ HDM SLIT‐Tablet is safe and well tolerated in patients with House Dust Mite allergic rhinitis with or without asthma: A “real‐life” French study

**DOI:** 10.1002/clt2.12129

**Published:** 2022-03-23

**Authors:** Pascal Demoly, Christophe Leroyer, Elie Serrano, Annelore Le Maux, Gabrielle Magnier, Antoine Chartier

**Affiliations:** ^1^ IDESP UMR UA11 INSERM—University of Montpellier Montpellier France; ^2^ Department of Pulmonology Division of Allergy Hôpital Arnaud de Villeneuve University Hospital of Montpellier University Montpellier Montpellier France; ^3^ Clinical Investigation Center CIC Inserm 1412 Hôpital Cavale Blanche University Hospital of Brest Brest France; ^4^ Department of ENT and Head and Neck Surgery Hôpital Larrey University Hospital of Toulouse Toulouse France; ^5^ Department of Medical ALK Courbevoie France

**Keywords:** Asthma, drug allergy, immunotherapy, Asthme, Immunothérapie, Traitement de l'allergie

## Abstract

**Background:**

The SQ House Dust Mite (HDM) SubLingual ImmunoTherapy (SLIT)‐Tablet (Acarizax) is the only allergen immunotherapy authorized by European regulatory authorities to treat HDM‐induced allergic asthma (AA) that is not well‐controlled by inhaled corticosteroids and associated with mild‐to‐severe HDM allergic rhinitis (AR). The aim of this study was to add evidence on the safety of the SQ HDM SLIT‐Tablet in patients with AR, alone or with AA, under real‐life conditions.

**Methods:**

This was a French “real‐life”, multicenter, non‐comparative, longitudinal, prospective study. It included patients initiating the SQ HDM SLIT‐Tablet for either persistent moderate‐to‐severe HDM AR or AA not well‐controlled by inhaled corticosteroids and associated with mild‐to‐severe HDM AR. Adverse Events (AEs) were collected at the first intake and throughout the study. Logistic regression was used to compare safety according to asthma control before treatment initiation.

**Results:**

Between May 09, 2018 and May 29, 2019, 1526 patients were enrolled at 185 sites and 1483 were included in the safety population (SAF). Of them, 33.6% had suspected clinical manifestations of AA. Asthma was uncontrolled for 18.2% of the patients, partially controlled for 27.9% and well‐controlled for 53.8%. Overall, 31.9% of the SAF patients experienced at least one AE. The percentage of patients with AEs was 29.9% among patients with AR alone and 35.9% among those with AA (*p* = 0.0193). No significant difference was observed in the rate of AE or SAE depending on asthma control at inclusion (2.2% of SAEs reported for patients with uncontrolled asthma, 1.4% for partly controlled and 1.1% for well‐controlled).

**Conclusions:**

The overall results indicate a good SQ HDM SLIT‐Tablet safety profile consistent with that reported in previous studies, regardless of asthma control.

## 1 INTRODUCTION

The prevalence of allergic rhinitis (AR) continues to increase worldwide, and it has been estimated that 500 million people suffer from AR and 300 million from asthma.^1^, ^2^ AR is thought to affect 24.5% of the French population sensitization to HDM is found in 49% of subjects with a clinical diagnosis of AR in western Europe.^3^ Among patients receiving allergen immunotherapy (AIT) for AR in France, 66.3% are treated with HDM allergenic extract.^4^ The association between AR and allergic asthma (AA) is well established^5^, ^6^, ^7^ and exposure to HDM allergens is associated with an increased risk of developing asthma and exacerbations of asthma.^8^, ^9^ In the European Community Respiratory Health Survey, the percentage of asthma attributable to HDM sensitization was 18.2% in the overall population and 12%–48% in various study centers in France.^10^ More broadly, with the change in western European lifestyles, citizens spend most time indoors and therefore are increasingly exposed to the indoor allergens known to be drivers of more severe phenotypes of AA.^11^ The only etiological treatment of HDM respiratory allergy is AIT. AIT can be administered either subcutaneously (SCIT) or sublingually (SLIT). In the recent EAACI guidelines,^12^ both routes of administration have shown efficacy in HDM AR and asthma, with a preference for the SQ HDM SLIT‐Tablet in terms of level of evidence for the decrease in severe exacerbations and ICS dose needed to maintain control. Importantly, if SCIT and SLIT can be used in controlled asthma, only this specific lyophilized formulation can be used in partly controlled asthma. The EAACI guidelines stress the importance of having more real‐life experience with the HDM tablet to support its use in partly controlled asthmatics. The Global Initiative for Asthma (GINA) 2021 guidelines specifically recommend considering HDM SLIT‐Tablet as add‐on therapy in HDM adult allergic asthmatics at risk of exacerbation in GINA steps 2–4. The SQ HDM SLIT‐Tablet (Acarizax) was approved by European regulatory authorities for the treatment of HDM‐induced persistent moderate‐to‐severe AR despite the use of symptom‐relieving medication in August 2015. It is the only immunotherapy authorized for the treatment of HDM‐induced AA not well‐controlled by inhaled corticosteroids and associated with mild‐to‐severe HDM AR in Europe. Several randomized controlled trials (RCTs) and two real‐life studies in Germany and Denmark/Sweden have evaluated the SQ HDM SLIT‐Tablet's safety and efficacy for the treatment of patients with HDM AR and/or AA.^13^, ^14^, ^15^, ^16^

This non‐interventional, open‐label and observational study aimed to add further evidence on the safety and tolerability of the SQ HDM SLIT‐Tablet in a real‐life setting in France in patients with AR alone or with AA.

## 2 MATERIALS AND METHODS

### 2.1 Study design and population

This was a “real‐life”, non‐interventional, French, multicenter, non‐comparative, longitudinal, prospective and descriptive study conducted between May 2018 and September 2020. Patients had to be aged between 18 and 65 years, with a clinical history and positive test of HDM sensitization (skin prick test and/or specific Immunoglobulin E [IgE]), and starting the SQ HDM SLIT‐Tablet for either of these two indications: persistent moderate‐to‐severe HDM AR despite the use of symptom‐relieving medication; or HDM AA not well‐controlled by inhaled corticosteroids and associated with mild‐to‐severe HDM AR. The decision to treat and the choice of indication had to be made before the patient entered the study and was left to the physician's discretion. Patients who had received any HDM immunotherapy in the 12 months prior to the study start were not included.

Patients were only included in the study after the decision to prescribe the SQ HDM SLIT‐Tablet had been taken and routine examinations and inquiries had been conducted and documented.

Physicians involved in the study comprised allergists, pulmonologists and ear, nose and throat (ENT) specialists. The study was proposed consecutively and exhaustively to all patients who met the eligibility criteria and had consulted the participating physicians during the 1‐year inclusion period, until the number of patients required for each group was obtained (competitive recruitment). The number of visits and examinations was at the physician's discretion, but patients were expected to attend up to four visits over a period of approximatively 12 months: two mandatory face‐to‐face visits at the start of the study, during which the SQ HDM SLIT‐Tablet was first administered and at the end; between these visits, two more were optional in line with the physician's real‐life practice to monitor tolerance and compliance. The last visits of the last patients were held more than a year after inclusion because of the COVID‐19 pandemic: there was a lockdown in France when these last visits should have taken place.

The symptom‐relieving medications against rhinitis, conjunctivitis or asthma taken during the last 12 months before the inclusion visit were recorded. The medical assessment of the rhinitis followed ARIA guidance and included the frequency and severity of symptoms: the AR could be classified as intermittent or persistent according to the frequency of symptoms per week, and as mild or moderate–severe depending on its severity. The control level of AR symptoms was assessed using the allergic rhinitis control test (ARCT) patient questionnaire.^17^ Asthma symptom control was assessed according to the GINA control score and the asthma control test (ACT) patient questionnaire. Although it was recommended to physicians that the SQ HDM SLIT‐Tablet be first administered under medical supervision, other modalities could be adopted, and therefore details of the first administration (during or after the first visit/supervised or unsupervised) were recorded by the investigator. Adverse events (AEs) and Serious Adverse Events (SAEs) were recorded at all planned and unscheduled visits. The severity and seriousness of the AE was judged by the investigator.

### 2.2 Objectives

The main objective of the study was to assess the tolerance and safety of the SQ HDM SLIT‐Tablet in a “real‐life” setting.

### 2.3 Sample size

The number of patients had been calculated independently for each physician group. To achieve sufficient accuracy (4.9%) for each specialty type, it was planned to include 500 patients per physician group (allergists, pulmonologists and ENT).

### 2.4 Statistical methods

Categorical variables were reported as frequency and percentage, while continuous variables were reported as mean, standard deviation (SD), median and interquartile range (IQR) values.

Two study populations were defined: the full analysis set (FAS), which included all patients who met all the eligibility criteria; and the SAFety (SAF) population, which comprised all patients who met all the eligibility criteria and received at least one dose of the SQ HDM SLIT‐Tablet.

Statistics describing baseline characteristics and safety analyses were generated on the SAF population. Results were provided overall and according to the clinical manifestation of allergy (AR patients with rhinitis only and AR patients with AA) and level of asthma control.

Groups were compared (AR vs. AA; physician specialty; study completed vs. premature discontinuation) using the chi^2^ test or Fisher's exact test (qualitative variables), and Student's *t*‐test (quantitative variables). The significance level was set at *p* < 0.05.

Comparisons between groups for safety analyses were made using a logistic model for items with data for 15 patients or more. The items considered for the regression analysis were patients with at least: one AE, one AE possibly related to the SQ HDM SLIT‐Tablet, one AE with severity = mild, one AE with severity = moderate, one AE with severity = severe, one AE leading to corrective treatment, one AE leading to temporary interruption of the SQ HDM SLIT‐Tablet, one AE leading to discontinuation of the SQ HDM SLIT‐Tablet, and one AE leading to no action taken on administration of the SQ HDM SLIT‐Tablet.

AEs were also analyzed according to the time of occurrence (on the day of first administration of the SQ HDM SLIT‐Tablet or during the entire study period).

#### 2.4.1 Ethical considerations

The study was conducted in accordance with Good Pharmacovigilance Practice guidelines and the Declaration of Helsinki (1964, and its amendments and subsequent clarifications) and the reference methodology MR‐003 granted by the French Data Protection Agency (CNIL). It was registered with the identification number 2017‐A02668‐45 and was approved by the Ethics Committee in October 2017. The patients provided written informed consent to participate.

## 3 RESULTS

### 3.1 Patients

Between May 09, 2018 and May 29, 2019, 1526 patients over the 12‐month period were enrolled at 185 French sites and 1494 were included in the FAS population. Thirty‐two (32) of the enrolled patients were excluded from the FAS population: 20 did not meet the selection criteria and the physician did not validate the data for 12. Eleven (11) patients from the FAS population did not take the SQ HDM SLIT‐Tablet and were excluded from the SAF population (*N* = 1483).

Of the 1483 patients included in the SAF population, 499 (33.6%) reported having clinical manifestations of AA at inclusion.

**FIGURE 1:**
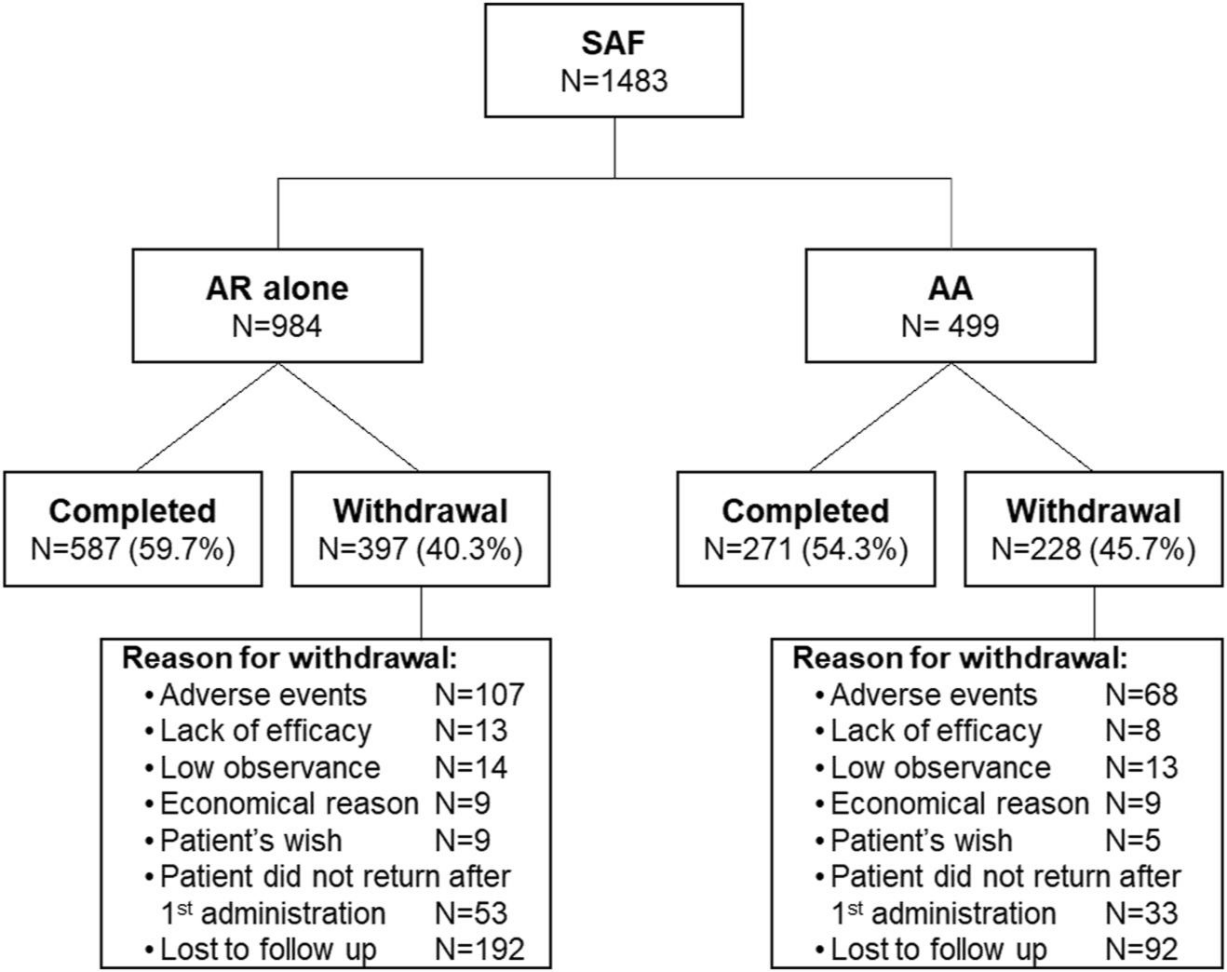
Patient flowchart. AA, allergic asthma; AR, allergic rhinitis; SAF, safety

At the end of the study, 858 (57.9%) patients had completed it according to the protocol (Figure 1). This percentage was similar across the different subpopulations (AR alone: 59.7%, AA: 54.3%). The physicians reported that 92.2% of patients who completed the study continued to take the SQ HDM SLIT‐Tablet after it ended. The occurrence of an AE was the reason for discontinuation for 175 patients (AR alone: 107, AA: 68). Patients discontinued the study (median [IQR]) 91.0 (28.5; 227.5) days after the first administration of the SQ HDM SLIT‐Tablet (AR alone: 83.0 [28.0; 210.0] days and AA: 119.0 [35.0; 266.0] days).

### 3.2 Baseline demographic and disease characteristics

Patient baseline characteristics are presented in Table 1. Over half of the patients were female (58.5%) and the mean (SD) age was 34.2 (11.5) years. The mean (SD) body mass index (BMI) was 24.1 (4.2) kg/m^2^: 24.0% of patients were overweight and 8.9% were obese. Most patients (79.9%) were non‐smokers.

**TABLE 1:** Demographic and disease characteristics at inclusion of patients in the SAF population

Parameter at inclusion	Statistics	AR alone	AA	Total
Total patients	*N*	984	499	1483
Age (years)	*N*	984	499	1483
	Mean (SD)	34.2 (11.6)	34.3 (11.2)	34.2 (11.5)
Gender	*N*	984	499	1483
Female	*n* (%)	581 (59.0)	286 (57.3)	867 (58.5)
Smoking habits	*N*	984	499	1483
Non‐smoker	*n* (%)	789 (80.2)	396 (79.4)	1185 (79.9)
Allergy history				
At least one respiratory allergy or sensitization (other than HDM)	*n* (%)	573 (58.2)^a^	370 (74.1)^a^	943 (63.6)
Disease characteristics				
Evaluation of rhinitis according to ARIA 2010	*N*	980	498	1478
Intermittent mild rhinitis	*n* (%)	41 (4.2)	41 (8.2)	82 (5.5)
Intermittent moderate–severe rhinitis	*n* (%)	44 (4.5)	44 (8.8)	88 (6.0)
Persistent mild rhinitis	*n* (%)	44 (4.5)	39 (7.8)	83 (5.6)
Persistent moderate–severe rhinitis	*n* (%)	851 (86.8)	374 (75.1)	1225 (82.9)
Severity of asthma according to GINA report 2017, *n* (%)	*N*		498	498
GINA—Step 1	*n* (%)	NA	148 (33.7)	148 (33.7)
GINA—Step 2	*n* (%)	NA	68 (13.7)	68 (13.7)
GINA—Step 3	*n* (%)	NA	211 (42.4)	211 (42.4)
GINA—Step 4	*n* (%)	NA	49 (9.8)	49 (9.8)
GINA—Step 5	*n* (%)	NA	2 (0.4)	2 (0.4)
Level of asthma control according to GINA report 2017, n (%)			494	494
Well controlled	*n* (%)	NA	266 (53.8)	266 (53.8)
Partly controlled	*n* (%)	NA	138 (27.9)	138 (27.9)
Uncontrolled	*n* (%)	NA	90 (18.2)	90 (18.2)

Abbreviations: BMI, body mass index; SD, standard deviation.

^a^ Chi^2^ test: *p* < 0.0001.

The percentage of patients with a sensitization other than HDM (tree, grass, weed, epithelia from furry animals, molds, food) was significantly greater among AA patients than among AR alone patients (76.2% vs. 60.0%; *p* < 0.0001). The three most frequent sensitizations overall and in all subgroups were grass (45.5% of the patients), epithelia from furry animals (42.3%) and tree (38.2%). Overall, 82.9% of the patients had persistent moderate–severe rhinitis according to ARIA 2010. The median (IQR) ARCT score was 17 (14–20) for the patients who completed the self‐questionnaire and 69.8% of them had uncontrolled AR (score <20). The percentage of patients with uncontrolled AR was 74.0% in the AR alone group and 61.4% in the AA group.

Among AA patients, asthma was uncontrolled for 18.2%, partially controlled for 27.9% and well‐controlled for 53.8% according to GINA report 2017. Most patients were taking step 3 treatment to control their asthma (42.4%), followed by step 1 (33.7%), step 2 (13.7%), step 4 (9.8%) and step 5 (0.4%).

Over the 12 months prior to the baseline visit, 4.0% of the AA patients experienced a mean (SD) number of 1.75 (1.29) severe asthma exacerbations (defined as a hospitalization >12 h or taking oral corticosteroids ≥3 days).

At inclusion, 83.9% of patients had never taken any HDM AIT and 16.1% reported having already taken HDM AIT but not in the 12 months before inclusion.

Baseline characteristics of patients who completed and prematurely discontinued the study have been compared and presented in Table S1. The two populations were comparable for most baseline characteristics (sex, BMI, polysensitization, time between first occurrence of HDM allergy and inclusion, rhinitis symptomatic medication, FEV1 results (%) of predicted value, asthma severity and control according to GINA report 2017, severe exacerbations of asthma in the last 12 months). Among patients who completed the study, there was a greater proportion of non‐smokers (*p* = 0.0277), fewer patients with asthma (*p* = 0.0488) and sleep disorders (*p* = 0.0008), and more patients with persistent moderate–severe rhinitis (*p* = 0.0215).

### 3.3 Modalities of the SQ HDM SLIT‐Tablet administration

Most (94.9%) patients took the first SQ HDM SLIT‐Tablet immediately (during the first visit) and did so under medical supervision (97.9%).

### 3.4 Safety

Overall, 31.9% of the SAF patients experienced 982 AEs over the course of the study; 872 of them were possibly related to the SQ HDM SLIT‐Tablet and were reported by 29.4% of patients.

The percentage of patients with at least one AE was 35.9% among those with AA and 29.9% among those with AR alone (*p* = 0.0193; Table 2).

**TABLE 2:** Adverse events by MedDRA preferred term occurring in ≥3% of patients

Parameter	Statistics	AR alone	AA	Total
Total patients	*N*	984	499	1483
Patients with at least one AE	*n* (%)	294 (29.9)	179 (35.9)	473 (31.9)
Patients with at least one:				
Throat irritation	*n* (%)	84 (8.5)	51 (10.2)	135 (9.1)
Oral pruritus	*n* (%)	81 (8.2)	46 (9.2)	127 (8.6)
Ear pruritus	*n* (%)	28 (2.9)	23 (4.6)	51 (3.4)

Abbreviation*:* AE, adverse event.

The percentage of patients with at least one AE according to physician specialty was 35.8% among the 984 patients included by allergists, 26.5% among the 389 patients included by pulmonologists and 16.4% among the 110 patients included by ENT (*p* < 0.0001).

Overall, 359 (24.2%) of the patients reported at least one mild AE, 162 (10.9%) at least one moderate AE and 49 (3.3%) at least one severe AE. Ten (10) patients reported 20 SAEs possibly related to the SQ HDM SLIT‐Tablet, five patients with AR alone and five with AA. Details of the SAEs are provided in Table S2. No anaphylactic shock or administration of adrenaline was reported. One patient with partly controlled asthma at inclusion experienced a moderate asthma exacerbation approximately 3 months after treatment initiation with no information on the delay after the SQ HDM SLIT‐Tablet intake. The patient was treated with short‐acting, selective beta‐2 adrenergic receptor agonists and the issue resolved. Another patient with HDM AR without AA at inclusion experienced hypoxemic pneumonia causing severe asthma exacerbation, leading to the patient's hospitalization 2 months after starting treatment with the SQ HDM SLIT‐Tablet. The patient recovered after being treated with an intravenous antibiotic course and intravenous corticosteroids. Another patient experienced lateral pharyngolaryngeal oedema, was treated with antihistamine and recovered. In all cases, the treatment with the SQ HDM SLIT‐Tablet was discontinued.

**FIGURE 2:**
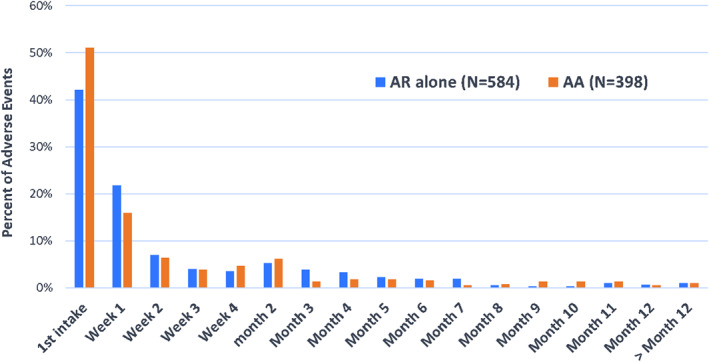
Distribution of time of occurrence of adverse events (possibly related or not) for patients with AR alone and AA (*N* = 982 AEs)

AEs occurred a median (IQR) 15.0 (3.0; 70.0) days after the first administration of the SQ HDM SLIT‐Tablet. Possibly related AEs occurred a median (IQR) 12.0 (2.0; 42.0) days after the first administration. About 45% of the AEs were reported at the time of first use of the SQ HDM SLIT‐Tablet. The number of AE possibly related or not occurrences decreased over time after the first administration of the SQ HDM SLIT‐Tablet (Figure 2) and a very similar trend was observed across the AR alone and AA subgroups.

The incidence of AEs depending on asthma symptom control according to GINA report 2017 is presented in Table 3. The percentage of patients to experience an AE was 36.8% among patients with well‐controlled asthma, 37.7% among those with partly controlled asthma and 32.2% among those with uncontrolled asthma. Most AEs in all subgroups were of mild severity and the percentage of AEs leading to discontinuation of the SQ HDM SLIT‐Tablet was similar across all subgroups (17.7% among patients with well‐controlled asthma, 21.7% among patients with partly controlled asthma and 17.8% among patients with uncontrolled asthma). The logistic regression analysis comparing the probability of AE occurrences (all AEs and AEs of different severity) in the three groups depending on asthma control showed no statistically significant differences (Table S3).

**TABLE 3:** Adverse events depending on asthma control according to GINA Score—SAF population with asthma as the clinical manifestation (*N* = 499)

Parameters	Statistics	Well controlled	Partly controlled	Uncontrolled
Total patients	*N*	266	138	90
Number of patients with at least				
One AE	*n* (%)	98 (36.8)	52 (37.7)	29 (32.2)
One AE possibly related to the SQ HDM SLIT‐Tablet	*n* (%)	93 (35.0)	47 (34.1)	26 (28.9)
One SAE	*n* (%)	3 (1.1)	2 (1.4)	2 (2.2)
One SAE possibly related to the SQ HDM SLIT‐Tablet	*n* (%)	1 (0.4)	2 (1.4)	2 (2.2)
One AE whose severity = ”mild”	*n* (%)	72 (27.1)	42 (30.4)	18 (20.0)
One AE whose severity = ”moderate”	*n* (%)	36 (13.5)	19 (13.8)	14 (15.6)
One AE whose severity = ”severe”	*n* (%)	8 (3.0)	2 (1.4)	6 (6.7)
One AE with corrective treatment(s)	*n* (%)	41 (15.4)	16 (11.6)	13 (14.4)
One AE leading to temporary interruption of the SQ HDM SLIT‐Tablet	*n* (%)	11 (4.1)	4 (2.9)	5 (5.6)
One AE leading to discontinuation of the SQ HDM SLIT‐Tablet	*n* (%)	47 (17.7)	30 (21.7)	16 (17.8)

*Note*: Percentages are based on all subjects from SAF excluding those with missing values. Patients with the “asthma control” parameter filled.

Abbreviations: AE, adverse event, SAE, serious adverse event.

The distribution of patients with at least one AE was similar across the GINA 2017 steps and a logistic regression model revealed that the probability of experiencing an AE, regardless of the severity, was not significantly associated with them (data not shown).

## 4 DISCUSSION

The primary aim of this study was to assess the safety and tolerability profile of the SQ HDM SLIT‐Tablet in adult patients over a period of 12 months. The patients could be included if they had a 12‐month washout period before the inclusion. While the washout period is generally 5 years for efficacy studies, Magnan et al. conducted a French phase IV, open‐label study showing that local AEs reappear at each annual reintroduction of AIT and consequently that 12‐month washout seems sufficient to assess safety and tolerability.^18^

Overall, 31.9% of patients experienced 982 AEs and 29.4% reported 872 AEs possibly related to the SQ HDM SLIT‐Tablet. Ten patients reported SAEs possibly related to the SQ HDM SLIT‐Tablet and 3.3% of patients reported at least one severe AE. No anaphylactic shock or administration of adrenaline was reported.

Two recent studies have reported data on the safety of the SQ HDM SLIT‐Tablet in a real‐life setting. Reiber et al.^13^ conducted a non‐interventional, open‐label study that included 1525 patients with AR alone (*N* = 1096) or with AA (*N* = 429) in Germany. Asthma at baseline was well‐controlled in 36.9% of the patients, partly controlled in 41.2% and uncontrolled in 22.0%. Sidenius et al.^14^ performed a real‐life non‐interventional study to investigate the safety profile, tolerability and outcome of the SQ HDM SLIT‐Tablet after 1 year of treatment in clinical practice among adults with HDM‐related AR with or without AA in Sweden and Denmark. The study included 198 patients, 58% had AR alone and 42% both AR and AA. Among patients with AA and AR, 52% had well‐controlled asthma, 26% partly controlled asthma and 22% uncontrolled asthma. The percentage of patients reporting an AE in this study was lower than that reported by Sidenius et al. (80%) and in RCTs,^15^, ^19^ but consistent with the occurrence reported by Reiber et al. (32.1%). In addition, by subgroups, the incidence of AEs was slightly higher among patients with AA (35.9%) compared to those with AR alone (29.9%), a trend observed in Reiber's study (41.5% of patients with AA and 28.6% of patients with AR alone).

Our results on the nature and severity of AEs also coincide with previous findings, and the most frequently reported AEs were throat irritation (9.1%), oral pruritus (8.6%) and ear pruritus (3.4%). The same events were the most common AEs reported by 20%–53% of patients in Sidenius et al.’s real‐life study.

The most common treatment‐related AEs occurred within the first few days of treatment with the SQ HDM SLIT‐Tablet and the percentage of patients experiencing the onset of an AE was greatest during the first week, as reported in previous studies. No systemic reaction was observed during the first intake in this broad real‐life patient population although they have been previously reported, albeit exceptionally.^20^ This possibility, as well as the importance of explaining the meaning of these frequent local side effects, justify the need to administer the first intake under medical supervision and provide close monitoring during the first 2–3 months.

The tolerance and safety profile observed in this study was also similar to the one observed in the EPIGRAM study, in which 504 adults and children received GRAZAX® for the treatment of grass pollen‐induced rhinitis and conjunctivitis under real‐life conditions.^21^ All but one patient had severe allergic rhino‐conjunctivitis. Adverse drug reactions were reported by 43% of the patients and serious adverse drug reactions (uvular edema, laryngeal edema and periodontitis) were reported in three patients. In the EPIGRAM study, asthma was associated with the allergic rhino‐conjunctivitis in 168 cases, but it was mostly controlled (89% of patients).

Subgroup analyses revealed no statistically significant difference in the risk of experiencing treatment‐related AEs depending on the asthma control. In addition, subjects with partly controlled or uncontrolled asthma were no more likely to experience AEs that would lead to discontinuation of the SQ HDM SLIT‐Tablet or to taking a corrective treatment than subjects with controlled asthma. These results are wholly consistent with those based on pooled safety data from P003 and MT‐04 trials reported by Emminger et al.^16^ The analysis included the results from two randomized placebo‐controlled trials to assess the safety and tolerability of the SQ HDM SLIT‐Tablet, which involved: (i) 834 adults with HDM AA not well controlled by inhaled corticosteroids and with HDM AR^22^; (ii) 992 adults with moderate‐to‐severe HDM AR despite the use of allergy pharmacotherapy and with or without asthma.^23^ In the pooled analysis, 50% of the patients treated with the SQ HDM SLIT‐Tablet reported at least one treatment‐related AE and 89.5% of them were mild. Most of the AEs occurred within the first few days of SLIT‐Tablet administration and resolved with continued treatment. The most common AEs were oral pruritus, as reported by 19% of patients, followed by throat irritation (15%) and mouth edema (12%).

There is little evidence on the safety of the SQ HDM SLIT‐Tablet for the treatment of patients with severe, unstable, uncontrolled asthma, and the presence of such a condition is a contraindication. This study, with 18.9% of patients having uncontrolled asthma according to the ACT scale, will enable complementing data from further studies and strengthen the picture on the SQ HDM SLIT‐Tablet's potential benefits and disadvantages for the indication.

This study had a very large sample size, ensuring representativeness of the French AR population with or without AA. However, potential limitations were the lack of quality control surrounding data collection and the loss of patients. All data must be considered, acknowledging that for some of the patients lost to follow‐up, no complete information was available at the time of their discontinuation.

## 5 CONCLUSIONS

In this study, the overall results indicate a good safety and tolerability profile of the SQ HDM SLIT‐Tablet, consistent with that reported in previous studies for the treatment of patients with HDM‐induced AR. In addition, with the favorable safety profile for the treatment of patients with AA regardless of control in real‐life settings, the SQ HDM SLIT‐Tablet represents a good treatment option for controlled or partly controlled asthmatic patients. Data from this study question the relevance of the current absolute contraindication in uncontrolled asthma.

## CONFLICT OF INTEREST

Pascal Demoly: Grant to hospital/association: ALK, STGR, GSK, AZ, Novartis, TFS, Sanofi.

Christophe Leroyer: Participation in boards, congresses and speakers: ALK, AstraZeneca, Chiesi, GSK, Menarini, Novartis, Pfizer, Sanofi.

Elie Serrano: Occasional consulting or expertise services and participation in expert boards for the pharmaceutical industry: ALK, Sanofi‐Aventis, Viatris, GSK, Nemera, Stallergenes, Zambon.

Annelore Le Maux: ALK corporate employee.

Gabrielle Magnier: Consultant to ALK.

Antoine Chartier: ALK corporate employee.

## AUTHOR CONTRIBUTIONS

**Pascal Demoly:** Conceptualization, Methodology, Validation, Visualization, Writing – original draft, Writing – review & editing. **Christophe Leroyer:** Conceptualization, Methodology, Validation, Visualization, Writing – original draft, Writing – review & editing. **Elie Serrano:** Conceptualization, Methodology, Validation, Visualization, Writing – original draft, Writing – review & editing. **Annelore Le Maux:** Data curation, Formal analysis, Methodology, Project administration, Supervision, Validation, Writing – original draft, Writing – review & editing. **Gabrielle Magnier:** Conceptualization, Data curation, Methodology, Project administration, Supervision, Validation, Writing – original draft, Writing – review & editing. **Antoine Chartier:** Conceptualization, Data curation, Methodology, Supervision, Validation, Visualization, Writing – original draft, Writing – review & editing.

## Supporting information

Supporting Information S1
