# Supplementary material for: The SQ HDM SLIT‐Tablet is safe and well tolerated in patients with House Dust Mite allergic rhinitis with or without asthma: A “real‐life” French study
Source: Clin Transl Allergy. 2022 Mar 23;12(3):e12129. doi: 10.1002/clt2.12129 (PMC8967264; doi:10.1002/clt2.12129)
Supplement: Supplementary file 1 — Supporting Information S1 [file CLT2-12-e12129-s001.docx]

**Table S1. Demographic and disease characteristics at inclusion of patients who completed the study in accordance with the protocol and who discontinued prematurely**

| **Parameters at inclusion** | **Statistic** | **Patients who completed**  **the study**  **(N=858)** | **Patients who discontinued prematurely**  **(N=625)** |
| --- | --- | --- | --- |
| **Patient characteristics** |  |  |  |
| Age (years) | N | 858 | 625 |
|  | Mean (SD) | 35.16 (11.64) | 32.92 |
|  | p-value^a^ | 0.0002 | |
|  |  |  |  |
| Gender | N | 858 | 625 |
| Male | n (%) | 370 (43.1) | 246 (39.4) |
| Female | n (%) | 488 (56.9) | 379 (60.6) |
|  | p-value^b^ | 0.1464 | |
|  |  |  |  |
| BMI (kg/m²) | N | 840 | 598 |
|  | Mean (SD) | 24.07 (4.00) | 24.18 (4.56) |
|  | p-value^a^ | 0.6213 | |
|  |  |  |  |
| Smoking habits | N | 858 | 625 |
| Non-smoker | n (%) | 705 (82.2) | 480 (76.8) |
| Previous smoker | n (%) | 72 (8.4) | 54 (8.6) |
| Active smoker (includes occasional smoking) | n (%) | 69 (8.0) | 73 (11.7) |
| Passive smoker | n (%) | 9 (1.0) | 11 (1.8 |
| Do not know | n (%) | 3 (0.3) | 7 (1.1) |
|  | p-value^c^ | 0.0277 | |
|  |  |  |  |
| **Allergy history** | **N** | **858** | **625** |
| At least one respiratory allergy | n (%) | 856 (99.8) | 620 (99.2) |
|  | p-value^c^ | 0.1394 | |
|  |  |  |  |
| At least one respiratory allergy (other than HDM allergy) | n (%) | 553 (64.5) | 417 (66.7) |
|  | p-value^b^ | 0.3646 | |
|  |  |  |  |
| At least one food allergy | n (%) | 47 (5.5) | 35 (5.6) |
|  | p-value^b^ | 0.9191 | |
|  |  |  |  |
| Time between first occurrence of HDM allergy and inclusion (years) | N | 856 | 623 |
|  | Mean (SD) | 7.20 (10.54) | 7.42 (9.92) |
|  | p-value^a^ | 0.6874 | |
|  |  |  |  |
| Clinical manifestation | N | 858 | 625 |
| Rhinitis | n (%) | 587 (68.4) | 397 (63.5) |
| Asthma associated | n (%) | 271 (31.6) | 228 (36.5) |
|  | p-value^b^ | 0.0488 | |
|  |  |  |  |
| FEV1 results (%) of predicted value, at baseline (among patients with Lung exploration) | N | 424 | 339 |
| <70 | n (%) | 11 (2.6) | 4 (1.2) |
| [70–80[ | n (%) | 28 (6.6) | 19 (5.6) |
| [80–90[ | n (%) | 64 (15.1) | 62 (18.3) |
| [90–100[ | n (%) | 123 (29.0) | 94 (27.7) |
| ≥100 | n (%) | 198 (46.7) | 160 (47.2) |
|  | p-value^b^ | 0.4771 | |
|  |  |  |  |
| **Sleep disorders** | **N** | **858** | **625** |
| Related to HDM allergy in the past month | n (%) | 323 (37.9) | 289 (46.5) |
|  | p-value^b^ | 0.0008 | |
|  |  |  |  |
| **Rhinitis symptomatic medication (antihistamines and/or nasal corticosteroids)** | **N** | **858** | **625** |
| During the last 12 months | n (%) | 817 (95.2) | 597 (95.5) |
|  | p-value^b^ | 0.7875 | |
| At the end of V1 | n (%) | 735 (85.7) | 545 (87.2) |
|  | p-value^b^ | 0.3956 | |
|  |  |  |  |
| **Evaluation of rhinitis according to ARIA 2010** | **N** | **857** | **621** |
| Intermittent mild rhinitis | n (%) | 45 (5.3) | 37 (6.0) |
| Intermittent moderate–severe rhinitis | n (%) | 42 (4.9) | 46 (7.4) |
| Persistent mild rhinitis | n (%) | 39 (4.6) | 44 (7.1) |
| Persistent moderate–severe rhinitis | n (%) | 731 (85.3) | 494 (79.5) |
|  | p-value^b^ | 0.0215 | |
|  |  |  |  |
| **Patients with clinical asthma** | **N** | **271** | **228** |
| Asthma severity according to GINA report 2017 | N | 271 | 227 |
| Step 1 | n (%) | 86 (31.7) | 82 (36.1) |
| Step 2 | n (%) | 31 (11.4) | 37 (16.3) |
| Step 3 | n (%) | 128 (47.2) | 83 (36.6) |
| Step 4 | n (%) | 25 (9.2) | 24 (10.6) |
| Step 5 | n (%) | 1 (0.4) | 1 (0.4) |
|  | p-value^b^ | 0.1709 | |
|  |  |  |  |
| Asthma control according to GINA report 2017 | N | 268 | 226 |
| Uncontrolled asthma | n (%) | 42 (15.7) | 48 (21.2) |
| Partially controlled asthma | n (%) | 80 (29.8) | 58 (25.7) |
| Well-controlled asthma | n (%) | 146 (54.5) | 120 (53.1) |
|  | p-value^b^ | 0.2347 | |
|  |  |  |  |
| Severe exacerbation(s) in the last 12 months | N | 271 | 227 |
| Yes | n (%) | 12 (4.4) | 8 (3.5) |
|  | p-value^b^ | 0.6089 | |

Abbreviations: BMI=Body mass index; SD=standard deviation; IQR=interquartile range; HDM=House dust mite; FEV1=Forced expiratory volume in 1 second; CRF=Case report form; ARIA=Allergic Rhinitis and its Impact on Asthma initiative; AE=Adverse Event

*^a^*Student’s t-test, *^b^*Chi2, *^c^*Fisher’s exact

**Table S2. Serious Adverse Events possibly related to the SQ HDM SLIT-Tablet by MedDRA preferred term – SAF population (N=1483)**

|  | **AR alone** | | **AA** |  | **Total** |
| --- | --- | --- | --- | --- | --- |
|  | N | Time after starting SLIT | N | Time after starting SLIT |  |
| **Number of patients, N** | **984** |  | **499** |  | **1483** |
| Patients with at least one SAE possibly related to treatment, n | 5 |  | 5 |  | 10 |
| ASTHMA | 1 | 2 months | 1 | 3 months | 2 |
| AUTOIMMUNE THYROIDITIS | 1 | 1 week | 0 |  | 1 |
| CHEST DISCOMFORT | 1 | 3 weeks | 0 |  | 1 |
| DYSPNEA | 1 | 3 weeks | 0 |  | 1 |
| EAR PRURITUS | 0 |  | 1 | 15 mins | 1 |
| FACE EDEMA | 0 |  | 1 | 1.5 months | 1 |
| HYPOXIA | 1 | 2 months | 0 |  | 1 |
| LUNG DISORDER | 1 | 2 months | 0 |  | 1 |
| MALAISE | 1 | 3 months | 0 |  | 1 |
| NASAL PRURITUS | 0 |  | 1 | 15 mins | 1 |
| ORAL PRURITUS | 0 |  | 1 | 15 mins | 1 |
| PALPITATIONS | 1 | 3 weeks | 0 |  | 1 |
| PHARYNGEAL OEDEMA | 0 |  | 1 | 2 months | 1 |
| RASH | 1 |  | 0 |  | 1 |
| RHINITIS | 0 |  | 1 | 5 mins | 1 |
| RHINITIS, ALLERGIC | 1 | 1 months | 0 |  | 1 |
| THROAT IRRITATION | 0 |  | 1 | 15 mins | 1 |
| THROAT TIGHTNESS | 1 | 3 weeks | 0 |  | 1 |
| TONGUE EDEMA | 0 |  | 1 | 1.5 months | 1 |

**Table S3. Probability of AE occurrence depending on asthma control – SAF population with asthma as the clinical manifestation (N=499)**

| **Parameters** | **N** | **OR [CI 95%]** | **P-value** |
| --- | --- | --- | --- |
| **At least one adverse event (AE)** | **N=494 *** |  | **0.6727** |
| Well controlled |  | 1.000 |  |
| Partly controlled |  | 1.036 [0.678 ; 1.585] | 0.8685 |
| Uncontrolled |  | 0.815 [0.491 ; 1.354] | 0.4294 |
|  |  |  |  |
| **At least one AE possibly related to the SQ HDM SLIT-Tablet** | **N=494 *** |  | **0.5696** |
| Well controlled |  | 1.000 |  |
| Partly controlled |  | 0.961 [0.623 ; 1.481] | 0.8562 |
| Uncontrolled |  | 0.756 [0.449 ; 1.272] | 0.2919 |
|  |  |  |  |
| **At least one AE unlikely related to the SQ HDM SLIT-Tablet** | **N=494 *** |  | **0.7576** |
| Well controlled |  | 1.000 |  |
| Partly controlled |  | 1.353 [0.610 ; 3.002] | 0.4565 |
| Uncontrolled |  | 1.116 [0.423 ; 2.945] | 0.8245 |
|  |  |  |  |
| **At least one AE whose severity = “Mild”** | **N=494 *** |  | **0.2201** |
| Well controlled |  | 1.000 |  |
| Partly controlled |  | 1.179 [0.750 ; 1.853] | 0.4759 |
| Uncontrolled |  | 0.674 [0.376 ; 1.207] | 0.1844 |
|  |  |  |  |
| **At least one AE whose severity = “Moderate”** | **N=494 *** |  | **0.8892** |
| Well controlled |  | 1.000 |  |
| Partly controlled |  | 1.020 [0.561 ; 1.855] | 0.948 |
| Uncontrolled |  | 1.177 [0.602 ; 2.299] | 0.6334 |
|  |  |  |  |
| **At least one AE whose severity = “Severe”** | **N=494 *** |  | **0.1149** |
| Well controlled |  | 1.000 |  |
| Partly controlled |  | 0.474 [0.099 ; 2.264] | 0.3496 |
| Uncontrolled |  | 2.303 [0.777 ; 6.829] | 0.1323 |
|  |  |  |  |
| **At least one AE with corrective treatment(s)** | **N=494 *** |  | **0.58** |
| Well controlled |  | 1.000 |  |
| Partly controlled |  | 0.720 [0.388 ; 1.336] | 0.2973 |
| Uncontrolled |  | 0.926 [0.471 ; 1.820] | 0.8246 |
|  |  |  |  |
| **At least one AE leading to discontinuation of the SQ HDM SLIT-Tablet** | **N=494 *** |  | **0.4617** |
| Well controlled |  | 1.000 |  |
| Partly controlled |  | 0.680 [0.368 ; 1.258] | 0.2195 |
| Uncontrolled |  | 0.955 [0.495 ; 1.843] | 0.8915 |
|  |  |  |  |
| **At least one AE leading to no action taken on the SQ HDM SLIT-Tablet** | **N=494 *** |  | **0.5132** |
| Well controlled |  | 1.000 |  |
| Partly controlled |  | 1.224 [0.766 ; 1.956] | 0.3968 |
| Uncontrolled |  | 0.862 [0.483 ; 1.540] | 0.6165 |

Percentages are based on all subjects from SAF, excluding those with missing values.

*Patients with the ‘asthma control’ parameter filled
